# Synthesis of a CFD Benchmark Exercise: Examining Fluid Flow and Residence-Time Distribution in a Water Model of Tundish

**DOI:** 10.3390/ma14185453

**Published:** 2021-09-21

**Authors:** Dong-Yuan Sheng

**Affiliations:** 1 Department of Materials Science and Engineering, Royal Institute of Technology, 10044 Stockholm, Sweden; shengdy@kth.se; Tel.: +46-8790-8467; 2 Westinghouse Electric Sweden AB, 72163 Västerås, Sweden

**Keywords:** CFD benchmark, meshing, turbulence model, discretization schemes, verification and validation (V&V), best practice guidelines (BPG)

## Abstract

Computational Fluid Dynamics (CFD) has become an indispensable tool that can potentially predict many phenomena of practical interest in the tundish. Model verification and validation (V&V) are essential parts of a CFD model development process if the models are to be used with sufficient confidence in real industrial tundish applications. The crucial aspects of CFD simulations in the tundish are addressed in this study, such as the selection of the turbulence models, meshing, boundary conditions, and selection of discretization schemes. A series of CFD benchmarking exercises are presented serving as selected examples of appropriate modelling strategies. A tundish database, initiated by German Steel Institute VDEH working group “Fluid Mechanics and Fluid Simulation”, was revisited with the aim of establishing a comprehensive set of best practice guidelines (BPG) in CFD simulations for tundish applications. These CFD benchmark exercises yield important results for the sensible application of CFD models and contribute to further improving the reliability of CFD applications in metallurgical reactors.

## 1. Introduction

Computational fluid dynamics (CFD) simulation was used to give insight into complex physical phenomena in a wide range of metallurgical reactors since it entered the research community. It has been adopted as a useful tool for the prediction of the flow behavior inside the tundish for several decades [1,2,3]. The main benefits of CFD applications in tundish include the following: (i) CFD modelling is wildly cheaper than making a costly prototype; (ii) engineers have the flexibility to quickly adjust CFD models, providing a better chance of ending up with an optimum product or solution; and (iii) CFD modelling allows the assessment of variables and data that cannot be accurately measured in experiments. [4,5].

The flow patterns in tundish with flow control devices (FCD) show three-dimensional behaviors which cannot be properly predicted by traditional one-dimensional analysis tools. Therefore, CFD codes can be applied to predict the flow phenomena in the tundish with better quality. CFD codes contain plenty of physical models for describing the turbulent flow, heat transfer, multi-phase interaction, and chemical reactions. In recent years, there have been a number of research works focusing on the CFD model application of the flow phenomena in tundish, such as residence time distribution [6,7,8], flow control device [9,10,11], inclusion behavior [12,13], and thermal status [14,15,16]. A series of CFD modelling studies have been recently published by the author through international collaborations [17,18,19,20,21]. The objective is to develop a modelling and simulation-based digital design methodology to optimize the tundish design and process parameters during the continuous casting process [22].

Model verification and validation (V&V) are essential parts of the model development process if CFD models will be used with sufficient confidence in industrial applications [23]. Quantitative V&V of CFD calculations are usually performed by comparing the model predictions against trustworthy data. A reliable assessment of the CFD model requires the calculations to be undertaken with complete control over the numerical errors and uncertainties to avoid erroneous conclusions. These requirements have prompted an initiative of the CFD benchmark exercise, which is carried out with the specific task of assessing the maturity of CFD codes, and to establish the best practice guidelines (BPGs) for the CFD applications [24].

The CFD benchmark exercise helps test different CFD codes, turbulence models, grid dependency, and boundary conditions. It gives a deeper understanding of how different numerical modelling method works in practice and discusses the reasonability of modelling results from one to another. The CFD benchmark exercise is commonly from a project with the collaboration in an organization. The issues raised in such benchmarks are the limited resources and coordination, lacking research continuity and sustainability. Therefore, the large international CFD benchmarks appeared in different industrial sectors, such as nuclear energy and automotive industry [25,26]. The objective is to assess the maturity of several CFD codes for the applications in specified area by a group of international specialists who can compile the various experimental and computational data, as well as the analytical solution data.

Blind CFD has been often used in the international benchmark exercises to examine currently available CFD models through comparison between results of independently performed CFD calculations and experimental results obtained in given geometries [27]. The experimental data were not released to the participants until at the end of the blind benchmark stage. Only the geometry, boundary conditions were supplied initially. After submitting the predictions to the benchmark coordinator, the experimental data were then released to the participants. This means that the participants could not fine-tune their CFD models to improve their results during the blind testing period. Blind test is an extremely useful tool to identify the variability in the CFD approach due to the differences in interpretation of high-level guidance and user variability.

In the metallurgical industry, a large number of CFD models have been applied to simulate the complex flow phenomena of the molten steel. However, very few international CFD benchmark database can be found in the literatures. Among the few published studies, Odenthal et al. presented the comprehensive CFD benchmark results of a single-strand continuous-casting tundish, initiated by German Steel Institute VDEH working group “Fluid Mechanics and Fluid Simulation”. This CFD benchmark was divided into two parts: (i) water model, named as Benchmark part I [28]; (ii) prototype (16-ton tundish), named as Benchmark part II [29]. In total, eleven members of the working group took part in the benchmark exercise, while ten members took part in the first part and nine members took part in the second part. The intention of Benchmark I was to perform the CFD simulation as blind test, for which only the tundish water model geometry, the volumetric flow rate at the shroud as well as the number of grid cells were defined. All other solution strategies and parameters were to be chosen freely by the participants. In addition to the flow structures, the RTD (Residence Time Distribution) curve was also calculated for the water model experiment. After this, the results were to be compared with each other and the models were critically assessed.

In this study, CFD benchmark exercises were performed as examples of appropriate modelling strategies. The benchmark exercises can be divided into two main groups: (i) comparison of CFD result between different modelling setup using the CFD software Star-CCM+; (ii) comparison of CFD results between current study and previous studies. The crucial aspects of CFD simulations in the tundish are addressed, such as the selection of the turbulence models, meshing, boundary conditions and selection of discretization schemes. The water model experimental data in Benchmark I was revisited with the aim of benchmarking a new applied CFD code (Star-CCM+), a new type of CFD mesh (polyhedral) and a high-order discretization scheme (third order). The objective is to establish a comprehensive set of best practice guidelines (BPG) in CFD simulations for tundish applications.

## 2. Model Description

### 2.1. Water Model

The water model is following the principle of geometry and dynamic similarity. The Reynolds criterion was used to calculate the volumetric flow rate for the steady-state simulation (see chapter dimension analysis in reference [28]). Reynolds number was calculated with the hydraulic diameter of the cross section of the tundish and the mean velocity through the cross section. The subject of the CFD benchmark is the water model, with the scale factor 1:1.7. The prototype is a 16-ton single-strand tundish used for the production of stainless steels. The experimental data used for the benchmark are results of residence time distribution and three-dimensional Laser Doppler Anemometry (LDA) measurements.

Once the water reached a normal operating level and was stabilized, the solute tracer was injected through the tundish inlet. One conductivity probe was used to record conductivity at the outlet of the tundish. The pulse stimulus–response technique was applied to obtain RTD curves. Afterwards, the time and the concentration were transformed to a dimensionless value to compare the obtained flow characteristics. The average values of three repetitions were used for data analysis.

The three-dimensional LDA measurements were made in the symmetry plane of the water model. It consists of 90 * 25 measuring points and 2000 samples have been measured at each point to give information about the velocity fluctuations of a flow. Other than velocity profile, it is also possible to indicate the flow turbulence by using turbulence intensity *Tu* which is defined as the ratio of the fluctuation velocity and the local flow velocity. The fluctuation velocity is obtained from turbulence kinetic energy *k*, as shown in Equation (1). The measuring system has an error whose amount can be estimated at approx. 5% [28].

(1) $$T_{u}=\frac{\sqrt{\frac{1}{3}\left(\bar{u^{\prime }{}^{2}}+\bar{u^{\prime }{}^{2}}+\bar{u^{\prime }{}^{2}}\right)}}{\left|\bar{u^{\prime }}\right|}=\frac{\sqrt{\frac{2}{3}k}}{\left|\bar{u^{\prime }}\right|}.$$

### 2.2. CFD Model

In this study, STAR-CCM + V.2020.1 was applied in the CFD modelling [30]. The assumptions made for the mathematical model are described below:

- The model is based on a 3D set of the Navier–Stokes equations;
- Water modelling is simulated under isothermal condition;
- Steady-state liquid flow is calculated;
- Reynolds averaged Navier–Stokes (RANS) turbulence models are used;
- The free surface is flat and is kept at a fixed level.

The calculation of single-phase incompressible flow is accomplished by solving the mass and momentum conservation equations. The equations solved in CFD code are written in a general form as [31]

(2) $$\rho \frac{\partial \bar{\emptyset }}{\partial t}+\rho \bar{u_{j}}\frac{\partial \bar{\emptyset }}{\partial x_{j}}-\frac{\partial }{\partial x_{j}}\left[\Gamma_{\emptyset ,eff}\frac{\partial \bar{\emptyset }}{\partial x_{j}}\right]=S_{\emptyset },$$

where ø represents the solved variable, Γ_ø,*eff*_ is the effective diffusion coefficient, *S*_ø_ is the source term, *x_j_* are the Cartesian coordinates, *u_j_* are the corresponding average velocity components, *t* is the time, and *ρ* is the density. The first term expresses the rate of change of ø with respect to time, the second term expresses the convection (transport due to fluid-flow), the third term expresses the diffusion (transport due to the variation of ø from point to point), and the fourth term expresses the source terms (associated with the creation or destruction of variable ø).

Equations (3) and (4) are the transport equations of continuous phase.

Continuity:

(3) $$\frac{\partial \left(\rho u_{j}\right)}{\partial x_{j}}=0.$$

Momentum:

(4) $$\rho u_{j}\frac{\partial u_{i}}{\partial x_{j}}=-\frac{\partial P}{\partial x_{i}}+\frac{\partial }{\partial x_{j}}\left[\left(\mu +\mu_{t}\right)\left\{\frac{\partial u_{i}}{\partial x_{j}}+\frac{\partial u_{j}}{\partial x_{i}}\right\}\right]+\rho g_{i}+S_{F},$$

where *ρ* is the density; *C_p_* is the heat capacity; *µ_t_* is the turbulent viscosity; *S_F_* represents the source term of momentum equation; *P* is the pressure.

Realizable *k*-*ε* model is given as below:

(5) $$\frac{\partial }{\partial t}\left(\rho k\right)+\frac{\partial }{\partial x_{j}}\left(\rho ku_{j}\right)=\frac{\partial }{\partial x_{j}}\left[\left(\mu +\frac{\mu_{t}}{\sigma_{k}}\right)\frac{\partial k}{\partial x_{j}}\right]+G_{k}+G_{b}-\rho \varepsilon -Y_{M}+S_{k},$$

(6) $$\frac{\partial }{\partial t}\left(\rho \varepsilon \right)+\frac{\partial }{\partial x_{j}}\left(\rho \varepsilon u_{j}\right)=\frac{\partial }{\partial x_{j}}\left[\left(\mu +\frac{\mu_{t}}{\sigma_{\varepsilon }}\right)\frac{\partial \varepsilon }{\partial x_{j}}\right]+\rho C_{1}S\varepsilon -\rho C_{2}\frac{\varepsilon^{2}}{k+\sqrt{v\varepsilon }}+C_{1\varepsilon }\frac{\varepsilon }{k}C_{3}G_{b}+S_{\varepsilon },$$

where *k* is the turbulent kinetic energy; *ε* is the turbulent energy dissipation rate; *µ* is the molecular viscosity; *µ_t_* is the turbulent viscosity; *G_k_* represents the generation of turbulent kinetic energy due to the mean velocity; *Y_M_* symbolizes the contribution of the fluctuating dilatation in compressible turbulence to the overall dissipation rate; υ is the kinematic viscosity; and *σ_k_* and *σ_ε_* are the turbulent Prandtl numbers for *k* and *ε*, respectively. The equations of other used turbulence model in this study can be found in reference [30].

Passive scalar equation is solved, including an instantaneous addition of the tracer at the inlet (E-curve). The passive scalar transport equations are solved at each time step once the fluid field is calculated.

(7) $$\rho \frac{\partial \bar{C}}{\partial t}+\rho \bar{u_{j}}\frac{\partial \bar{C}}{\partial x_{j}}-\frac{\partial }{\partial x_{j}}\left[D_{eff}\frac{\partial \bar{C}}{\partial x_{j}}\right]=0,$$

where *D_eff_* is the effective diffusivity, *t* is the time, and C is the concentration. The velocity field is solved obtained from a steady-state simulation and remained constant during the calculation of the passive scalar.

### 2.3. Geometry and Mesh

**Figure 1:**
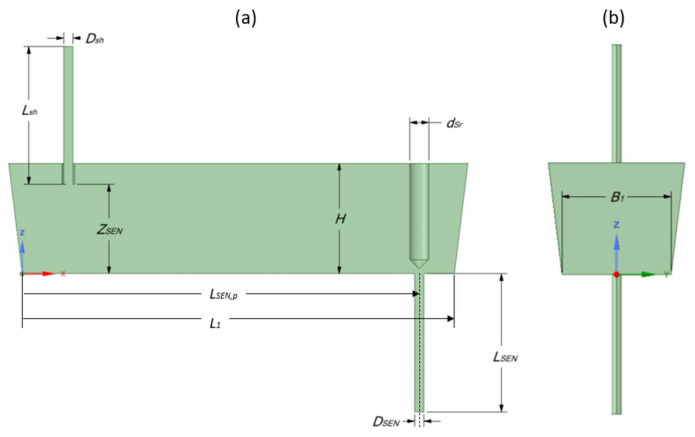
Geometry of the tundish (**a**) front view (**b**) side view.

The geometrical dimensions of a single-strand 16-ton slab tundish, used for the production of stainless steels, are illustrated in Figure 1. The water model test facilities can be operated in a closed or open water cycle and basically consist of shroud, stopper rod, SEN, pump, magneto-inductive flow meter, and flow control valve [32]. The shroud and stopper rod are adjustable to reveal the influence of asymmetrical flow effects. The geometry of the tundish is given in Table 1.

**Figure 2:**
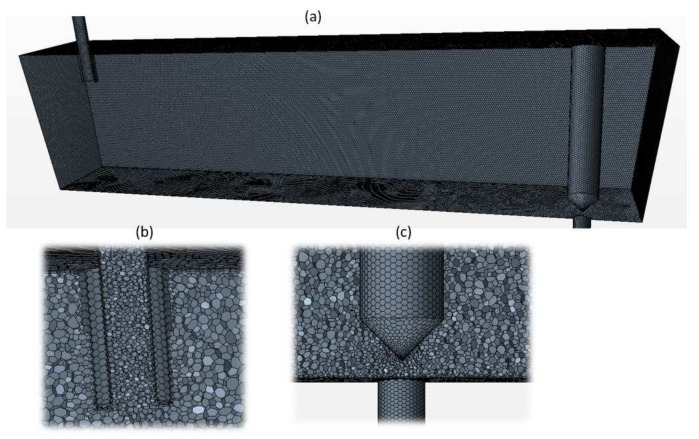
CFD Mesh, (**a**) full water model tundish, (**b**) zoom in of inlet part, (**c**) zoom in of stopper rod and outlet.

To create the geometry for CFD calculation, the first step was to build a 3D-CAD model. The volume mesh was generated in Star-CCM+ using the polyhedral mesh and prism layer meshing options, shown in Figure 2. Three prism layers were generated next to all the walls. Six different CFD mesh sizes were investigated. The surface mesh was generated first. Then the volume mesh was built based on the surface mesh by adjusting the growth rate. The input parameters for the CFD simulations are listed in Table 1. To save the computing time, a half tundish was simulated through its symmetry plane.

### 2.4. Numerical Modelling Details and Boundary Conditions

No-slip conditions are applied on all solid surfaces for the liquid steel phase. A constant inlet velocity (2.92 m/s) is used. At the tundish outlet, the outflow boundary condition is applied. A wall function is used to bridge the viscous sub-layer and to provide the near-wall boundary conditions for the average flow and the turbulence transport equations. The wall conditions are connected by means of empirical formulae to the first grid node close to the solid surfaces. At the top surface, no-slip wall function is used, according the modelling setup from user 3 and user 10 in Benchmark I [28]. The applied initial conditions are the default settings in the Star-CCM+ software. The details of setting up the CFD model and numerical solution procedure can be found in reference [20,21].

The discretized equations are solved in a segregated manner with the semi-implicit method for the pressure-linked equations (SIMPLE) algorithm. Three different discretization scheme (1st, 2nd, and 3rd order) are used to calculate the convective flux in the momentum equations. The solution is judged to be converged when the residuals of all flow variables are less than 1 × 10^−4^, together with the stability of the velocity and the turbulence at the key monitor points. The flow fields are calculated in steady state. The transient calculation is used to solve the passive scalar equation for the residence-time distribution calculations. The under-relaxation parameters of flow calculations for the pressure, the velocity and the turbulence are 0.3, 0.7, and 0.8, respectively.

## 3. Results

### 3.1. Reference Data of Fluid Flow (Water Model)

**Figure 3:**
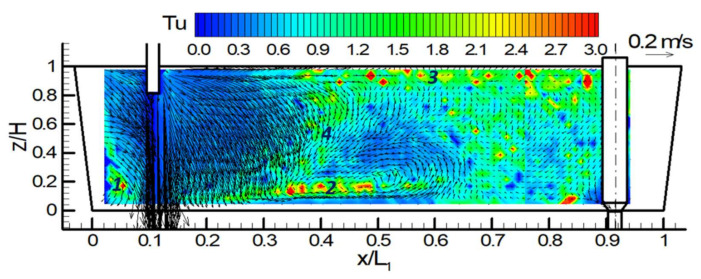
Measured velocity and turbulence intensity distribution at the central longitudinal plane in the water model of tundish (geometry scale 1:1.7) [28].

The reference data of fluid flow is the result of three-dimensional Laser Doppler Anemometry (LDA) measurements in the water model of tundish. Figure 3 illustrates the 3D-LDA results for the steady-state [28]. The incoming flow jet enters the tundish through the shroud. The high momentum jet flows towards the front and sidewalls once it reaches the tundish bottom. Four significant turbulence regions can be identified based on the contour color from Figure 2. They are marked by 1, 2, 3, and 4 in the figure.

(1) The edge of the tundish near the shroud (0 < x/L_1_ < 0.08);
(2) A thin horizontal region along the tundish bottom (0.2 < x/L_1_ < 0.76);
(3) A horizontal region just below the free surface (0.38 < x/L_1_ < 0.76);
(4) An inclined vertical region between free surface and bottom (0.2 < x/L_1_ < 0.45).

Turbulence is produced in regions of high velocity gradients even though the mean velocity is low. At the central longitudinal plane, it formed a counter-clockwise vortex near the center of tundish length and close to the tundish bottom. The vortex center located at x/L_1_ = 0.52 and z/H = 0.2. The maximum backflow velocity on this plane is approximately 0.07 m/s (u) with a position of x/L_1_ = 0.49 and z/H = 0.39. These will be used as a criterion to validate the CFD modelling.

### 3.2. Benchmark of Fluid Flow (Current Participant)

The sensitivity analysis of CFD model was performed with the focus on mesh size, turbulence model, and discretization scheme.

#### 3.2.1. Mesh Size

Mesh size should be small enough to be able to capture the flow characteristics, especially for the region with high turbulence. A finer mesh causes a longer computation time. Thus, it should also consider the computation capacity when selecting the mesh size for the CFD model. Here, CFD models with mesh size varied from 0.0045 to 0.007 m are studied and compared. The total mesh number of CFD model can be found in Table 2.

**Figure 4:**
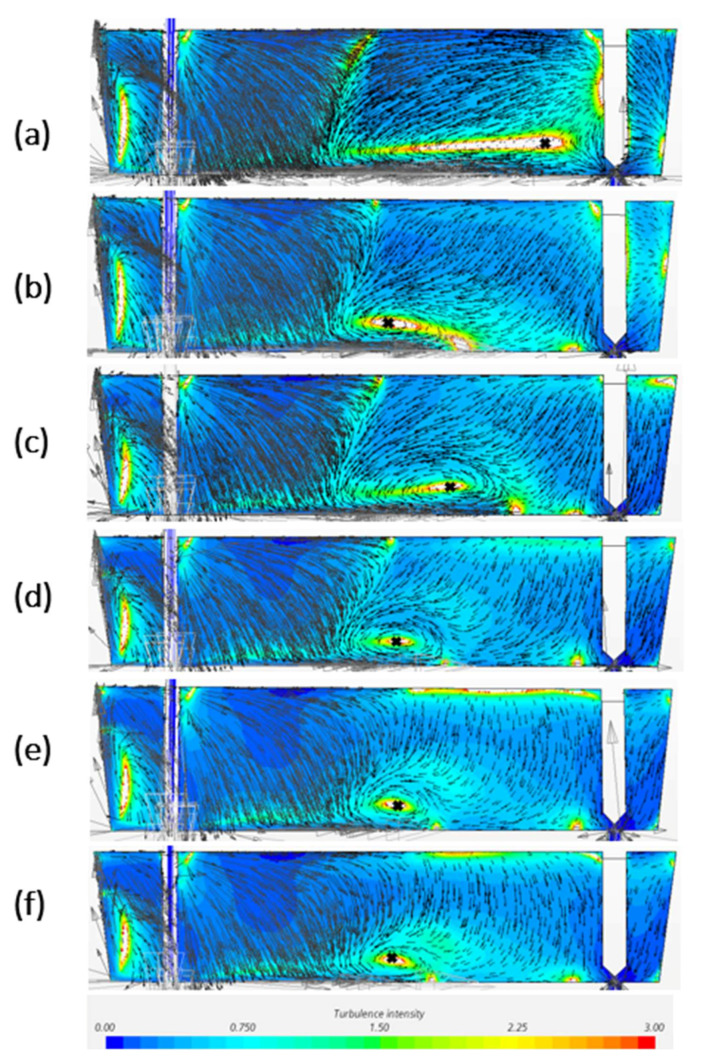
Turbulence intensity and velocity profile of the CFD models with mesh size of (**a**) 0.0045 m, (**b**) 0.005 m, (**c**) 0.0055 m, (**d**) 0.006 m, (**e**) 0.0065 m, (**f**) 0.007 m on symmetry plane.

Figure 4 is the predicted profiles of turbulence intensity and flow velocity on central plane by CFD models with different mesh size. In comparison with Figure 3, all the models can predict the turbulence zone in the tundish edge near the shroud. The thin horizontal recirculation region closed to the tundish bottom are described among the models; however, the recirculation shape and the center of recirculation on the central plane displayed differently. With the decreased mesh size, the recirculation center has a tendency to move towards the direction of the SEN. In addition, the inclined turbulence zone between free surface and bottom can be easier predicted by finer meshes. A bigger mesh such as 0.0065 m and 0.007 m would be difficult to predict this inclined turbulence region.

In comparison of CFD modelling results in Figure 4 with experiment measurement in Figure 3, the CFD models with mesh size 0.0055 and 0.006 m have been considered as a better prediction. The model with 0.006 m mesh shows the best prediction of all the four high turbulence regions, as well as a rather good agreement of vortex center (x/L_1_ = 0.5). However, when it comes to the recirculation zone shape, 0.0055 m gives a recirculation area between 0.35 < x/L_1_ < 0.55, which is better in agreement with experiment measurements than the prediction from 0.006 m model.

**Figure 5:**
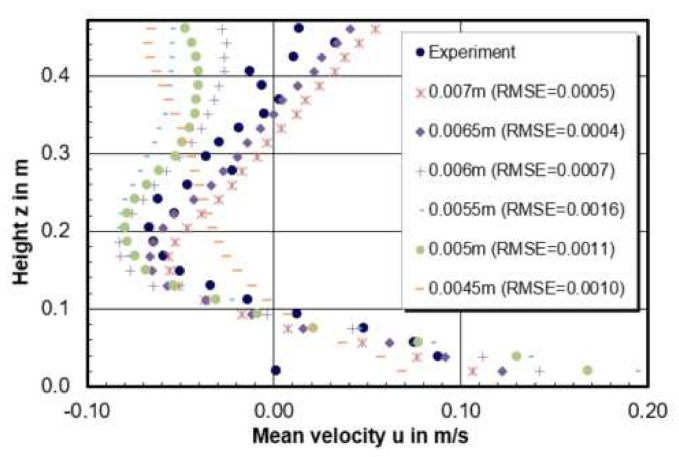
Horizontal velocity (u) through the recirculation center of CFD models with mesh size of (**a**) 0.0045 m, (**b**) 0.005 m, (**c**) 0.0055 m, (**d**) 0.006 m, (**e**) 0.0065 m, (**f**) 0.007 m.

Figure 5 gives the velocity profiles along the recirculation center on the central plane of the model. In total, 24 measurement points were selected to get the horizontal velocity (u) along the vortex center. A simple metric Root Mean Square Error (RMSE) is chosen to compare the relative quality of CFD calculations. *RMSE* is defined in Equation (8) and *n* is the total number of experimental points. The lower the value for *RMSE*, the better of CFD model is for the prediction.

(8) $$RMSE=\sqrt{\frac{1}{n}\times \sum {\left(experiment-predict\right)}^{2}}.$$

According to the result, the ranking of the CFD modelling quality in terms of horizontal velocity through the recirculation center is 0.0065 m > 0.0007 m > 0.0006 m > 0.0045 m > 0.005 m > 0.0055 m. The best prediction model is the model with a mesh size of 0.0065 m which has the lowest RMSE value of 0.0004. In addition, it can be seen from Figure 5 that the smaller mesh size model has tendency to drive the flow to the negative horizontal direction, especially near the free surface. When the negative horizontal flow meets with the positive horizontal flow coming from the shroud, it is easily forming a high turbulence intensity zone due to the high velocity gradient. This can explain why it is easier to show the inclined high turbulence region between free surface and the tundish bottom for smaller mesh in Figure 4.

**Figure 6:**
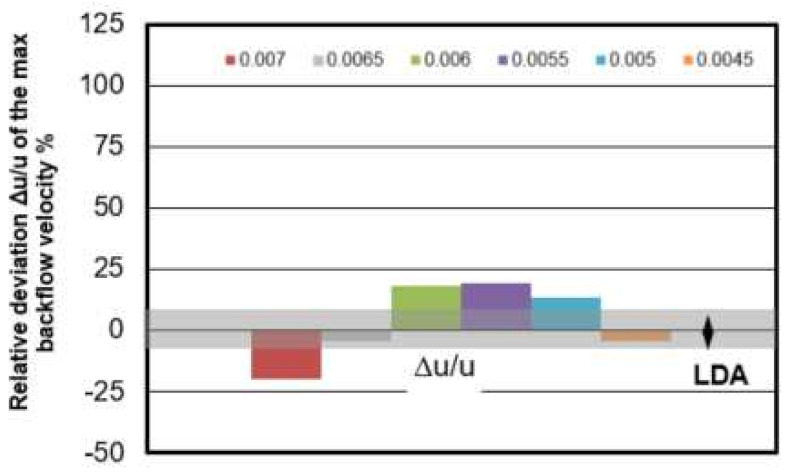
Relative deviation Δu/u of the maximum backflow velocity between CFD simulation and measurements.

The relative deviation of computed maximum backflow velocity in vortex region with the measured maximum back flow velocity value u = 0.007 m/s is shown in Figure 6. The measuring error of LDA mean velocity value is around ±7% which is marked as gray bar in Figure 6. The deviation in this mesh size study is between −20% < Δu/u < 19%. The smallest deviation is found in the mesh of 0.0045 m which is −4%.

**Figure 7:**
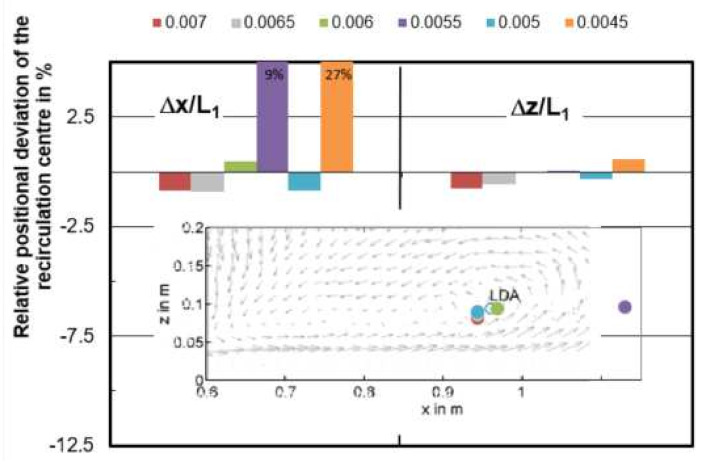
Relative positional deviation Δx/L_1_ and Δz/L_1_ of the recirculation center from CFD models with varied mesh size (y/L_1_ = 0).

Figure 7 gives the relative position deviation Δx/L_1_ and Δz/L_1_ of the vortex center with reference to the measured center through LDA. Among the models with varied mesh size, the smallest deviation can be found in the mesh of 0.006 m which is 0.5% and −0.1% for Δx/L_1_ and Δz/L_1_, respectively. When the mesh size is 0.0045 m and 0.0055 m, the simulated vortex center differs quite a lot from the measured position, the center of 0.0045 m is even out of the figure boundary.

#### 3.2.2. Turbulence Model

Selection of a proper turbulence model is important for conducting modelling performance evaluation of tundish system. Reynolds averaged Navier–Stokes (RANS) based turbulence models were evaluated including Eddy viscosity models (EVM) and a Reynolds Stress Model (RSM). The concept of eddy viscosity has gained the most attention among numbers of tundish CFD investigations in a few decades because they have in general better balance between simulation accuracy and computing resource. The EVM models characterized the flow field by solving two extra transport equations. The most widely used k-ε model group (standard, realizable, two-layer, low-Reynolds number, elliptic blending, lag elliptic blending, V2F, etc.) is the modification of the standard k-ε model developed by Lauder and Spalding in 1972 [34]. The k-ω model is also belonging to the two-equation EVM. Reynolds stress transport model is more advantageous in complex turbulence flow with a large streamline curvature and swirl but it can be computational intensive and difficult to converge compared to EVM. Six RANS turbulence models are compared and discussed in this work, including

- Standard k-ε (SKE);
- Realizable k-ε (RKE);
- V2F k-ε (VKE);
- Elliptic blending k-ε(EBKE);
- Shear stress transport k-ω (SKW);
- Reynolds stress models (RSM).

**Figure 8:**
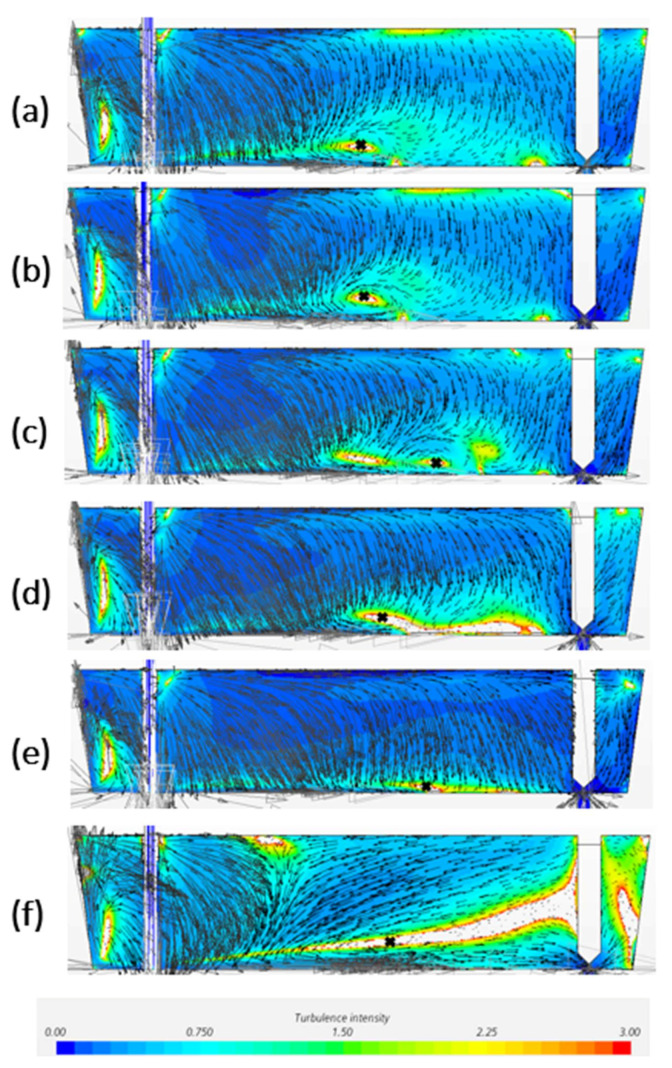
Velocity and turbulence intensity of water model tundish (mesh size: 0.006 m) with turbulence model of (**a**) SKE, (**b**) RKE, (**c**) VKE, (**d**) EBKE, (**e**) SKW, (**f**) RSM on symmetry plane.

**Figure 9:**
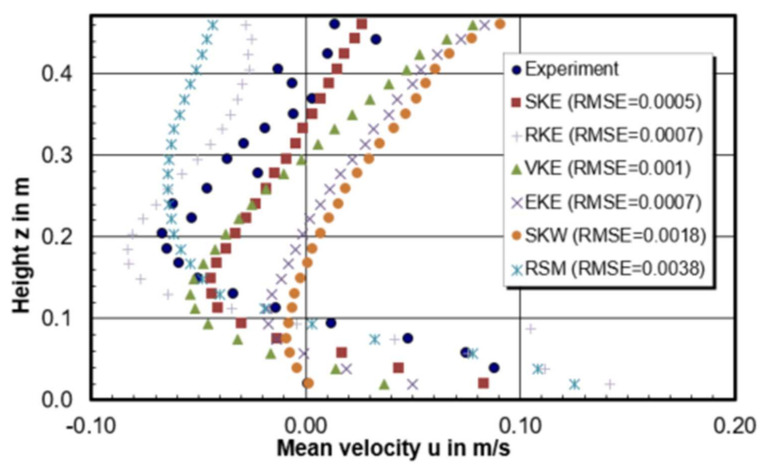
Comparison of horizontal u velocity in a vertical section through the center of the circulation region and root mean squared error (RMSE) between CFD modelling and experiment (mesh size: 0.006 m).

**Figure 10:**
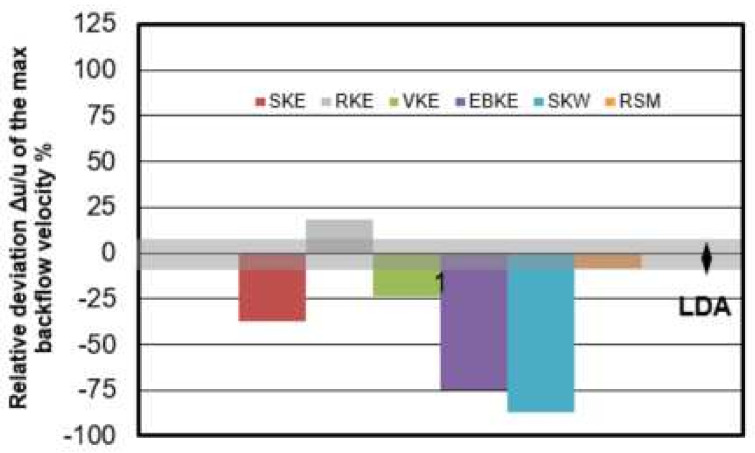
Relative deviation Δu/u of the maximum backflow velocity between CFD models with different turbulence model.

**Figure 11:**
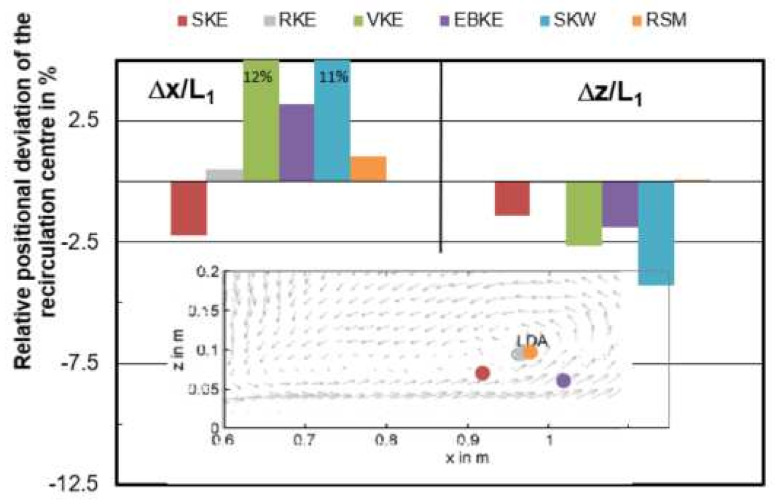
Relative positional deviation Δx/L_1_ and Δz/L_1_ of the recirculation center from CFD models with varied turbulence model (y/L_1_ = 0).

Velocity and turbulence intensity of water model tundish with different turbulence models are presented in Figure 8. It shows that k-ω and RSM models are failed to predict the recirculation zone near the middle bottom of tundish. When comparing the horizontal u velocity through the recirculation zone, RSM turbulence model seems to obtain a higher turbulence intensity flow in general. As suggested by Figure 9, when comparing the simulated horizontal velocity through the vertical recirculation zone and experimental data, the modelling quality is SKE > RKE = EKE > VKE > SKW > RSM. However, in Figure 10 RSM turbulence model indicated the best prediction of maximum back flow velocity. This can be explained by that the selection of recirculation area is kind of subjective which can influence the backflow velocity as well. In Figure 11, the best prediction of relative positional deviation of the vortex center is the model with realizable k-ε model, the deviation percentage on the horizontal vertical direction is 0.5% and −0.1%, respectively.

#### 3.2.3. Discretization Schemes

The selection of discretization schemes for convection can affect the modelling convergence and accuracy. Three convection discretization schemes are compared in this work. They are

- The first-order upwind scheme (1st order);
- The second-order upwind scheme (2nd order);
- The third-order MUSCL (3rd order).

**Figure 12:**
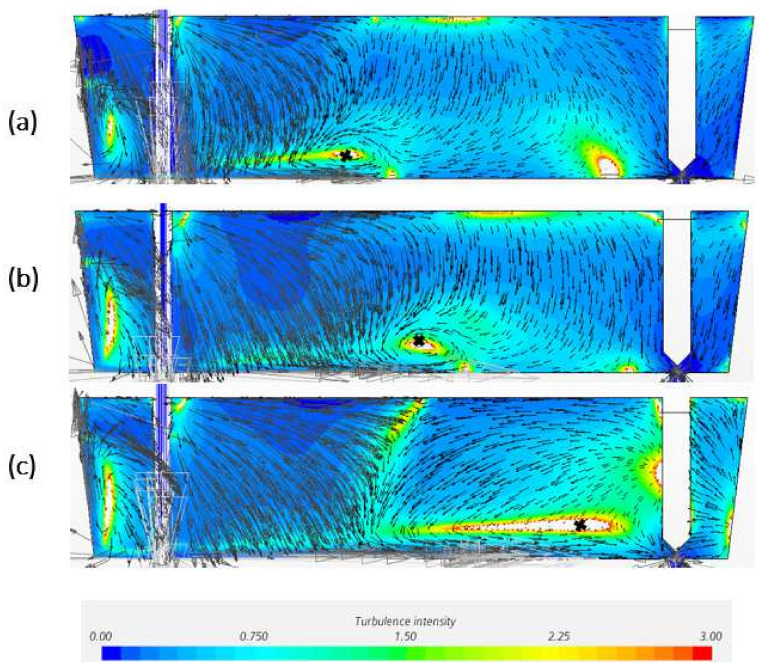
Velocity and turbulence intensity of water model tundish (mesh size: 0.006 m, realizable k-ε) with (**a**) first order upwind, (**b**) second order upwind, (**c**) third order MUSCL discretization method on symmetry plane.

**Figure 13:**
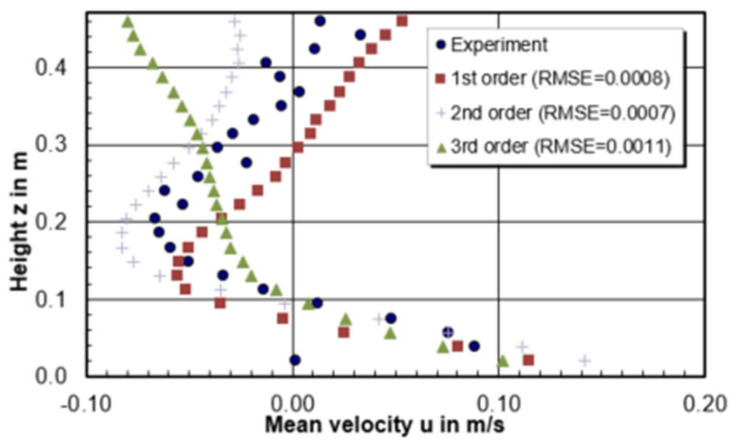
Comparison of horizontal u velocity in a vertical section through the center of the circulation region and RMSE between CFD modelling and experiment.

In the CFD software STAR-CCM+, the default setting is the second order. For the presented simulations, the second order discretization method shows the best results compared with experimental results based on Figure 12 and Figure 13. The model using first order and third order failed to predict the flow pattern. The recirculation zone is appearing closed to the inlet zone while the recirculation zone is near the SEN. In general, low-order discretization yields better convergence and less accurate results, while the high-order scheme may bring greater accuracy as well as numerical difficulties and instabilities. Most often, the second upwind scheme is applied in industrial application. Based on the above results, second order discretization method predicts a rather reasonable recirculation zone position where the vortex center is closed to the center of tundish length.

### 3.3. Benchmark of Fluid Flow (All Participants)

Current work is noted as User 12 in order to correspond with the published Benchmark I [12]. The detailed model settings for all participants are described in Table 3. Although the model sensitivity studies have been performed in Section 3.2, only one calculation was selected for the summary of all participants’ contribution. The selected model parameters by User 12 are (i) mesh size, 0.006 m; (ii) realizable k-ε turbulence model; and (iii) 2nd order upwind discretization scheme.

**Figure 14:**
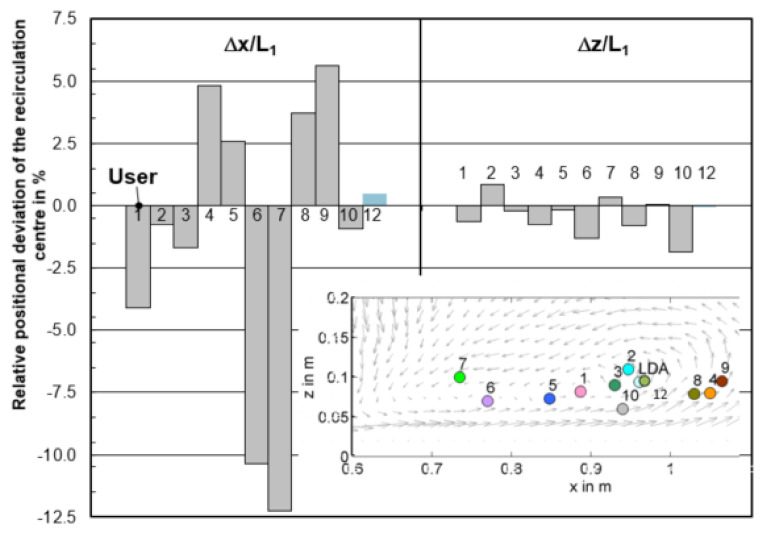
Relative positional deviation Δx/L_1_ and Δz/L_1_ of the recirculation center from CFD models among users (y/L_1_ = 0).

**Figure 15:**
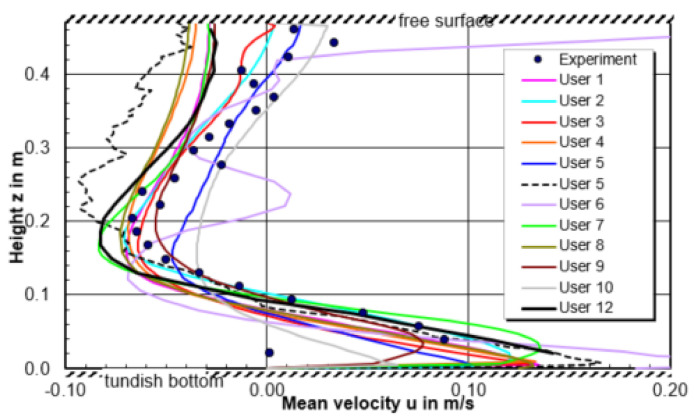
Comparison of horizontal u velocity in a vertical section through the center of the circulation region between users and experiment.

The predicted recirculation center among all the participants on the central plane is shown in Figure 14. In horizontal direction, the relative positional variation is −12% < Δx/L_1_ < 5%, in vertical direction it is even as little as −2% < Δz/L_1_ < 1%. It indicates that all participants safely predicted the vortex center. The horizontal u velocity is plotted as a function of vertical z-component, shown in Figure 15. It can be seen that the main variance of velocity among users is above the top half liquid height (z > 0.2 m). Under the free surface, the velocities predicted by user 2, 3, 5, 6, and 10 velocity show quite well agreement with measurements. This depends on the boundary condition used at the top surface. In general, the symmetry boundary condition at top surface shows a relative worse prediction. In the low half liquid height (z > 0.2 m), it shows a good agreement between the CFD predictions and measurements.

**Figure 16:**
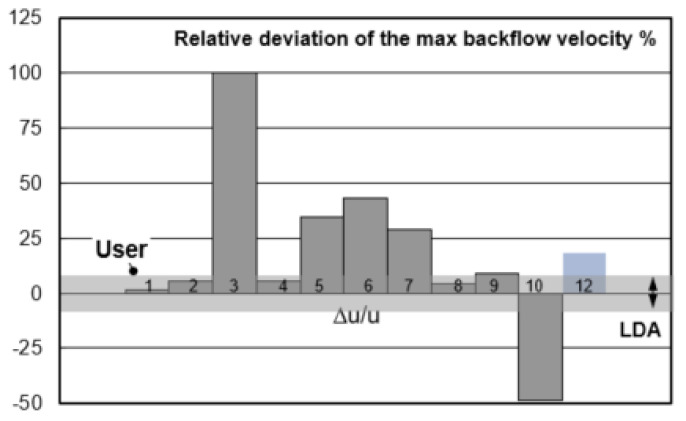
Relative deviation Δu/u of the maximum backflow velocity between CFD models among users.

Figure 16 shows the relative deviation of maximum backflow velocity in the recirculation area. The error tolerance of LDA is ±7% and marked as gray bar. User 1, 2, 4, and 8 predicted the maximum backflow velocity within the error tolerance. It is recommended to use the realizable k-ε model or RSM model and a high order discretization method. The result of User 12 using realizable k-ε and 2nd order discretization scheme seems not as good as expected here for the prediction of maximum backflow velocity. Differently, the result of User 12 shows a good prediction of recirculation center in Figure 14.

### 3.4. Benchmark of Residence-Time Distribution (All Participants)

**Figure 17:**
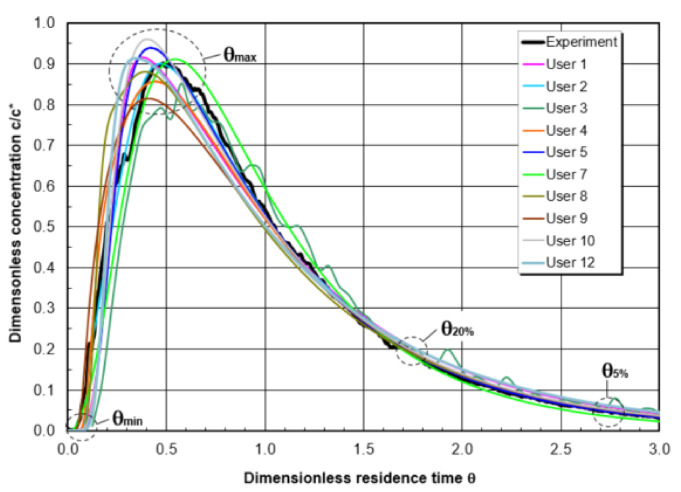
RTD curves among users.

**Figure 18:**
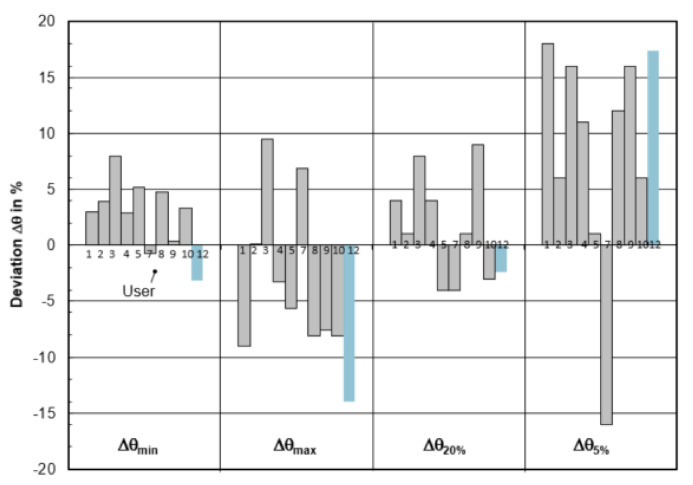
Relative deviation of the calculated RTD data Δθ_min_, Δθ_max_, Δθ_20%_, Δθ_5%_.

Figure 17 exhibits the comparison between the measured and computed RTD curves. The theoretical residence time in the water model is 126 s. The selected criteria are (i) the minimum residence time θ_min;_ (ii) the maximum concentration time θ_max_; (iii) the time θ_20%_ (RTD curve dropped to 20% of the maximum level); and the time θ_5%_ (RTD curve dropped to 5% of the maximum level), respectively. All the CFD simulated RTD curves agree quite well with the measured RTD curve. It indicates that the CFD modelling can be an effective tool for the design of flow control device in tundish, using the RTD analysis as an important criterion. As shown in Figure 18, it is difficult to draw some relationship between the deviations and the selected CFD models. This may be due to the different time-step size Δt used for the computation of the RTD curve. This value was not reported in Benchmark I. In the current study (User 12), the time-step Δt is set as one second.

## 4. Conclusions

CFD simulations of the sensitivity studies have been performed based on the different modelling setting including mesh size, turbulence model, and discretization scheme. The presented modelling results show that even for a simple single-phase flow, the predicted flow pattern in tundish can vary a lot when changing only one modelling parameter. This variance implies the risk of wrong choice, which can be mostly avoid by the comparison with the experimental data and by the learning process of the similar problems. As an individual CFD user, own methodology and guideline should be set up to select the modelling parameters. For instance, in current study, a recommended modelling setup for tundish flow simulation is the mesh size 0.006 m, realizable k-ε turbulence model and second order upwind discretization scheme.

From the flow results of all benchmark participants, a quite large performance variance of CFD model was observed. Realizable k-ε model is good at predicting the turbulence zone in the tundish but may be not successful in simulating the maximum backflow velocity. It is necessary to conduct different experiment measurements to validate the modelling approach from different aspects. Furthermore, this brings the question of how to define a meaningful measure of the accuracy of a CFD simulation model.

The flow in tundish represents an interesting example of the coexistence of high turbulent regions (near the inlet and outlet) and relative quiescent regions. From the CFD modelling point of view, it is a difficult task to simulate the system using RANS model if a more detailed local turbulence structure needs to be investigated. Following with the development of computational capacity, LES or DNS can be a useful method to capture the local flow phenomena in detail in the future work.

The CFD simulated RTD curves of all benchmark participants agree quite well with the measured RTD curve. It indicates that the CFD modelling can be an effective tool for the design of flow control device in the tundish when the RTD analysis can be considered as a design criterion.

To sum up, it is necessary to perform a continuous CFD benchmark exercise in the tundish. On one hand, it provides the opportunity to the individual CFD user with the comparison of the high-quality experiment data. By the validation with a high standard experiment data, the prediction accuracy of CFD model can be improved. On the other hand, the individual user can also compare the modelling results with other participants who might use different software tools and different methodologies. There are many experiences in the theoretical study and practical practice that can be shared by a large user group, especially in the development of the large benchmark database for the CFD V&V in the metallurgical applications.

## Figures and Tables

**Table 1:** Geometry and process data of single-strand tundish [28,29,33].

Notations		Unit	Tundish	
			Prototype	Water Model
Liquid flow volume in the tundish	*V*	m^3^	2.275	0.463
Length of the tundish bottom	*L_1_*	m	3.14	1.847
Width of the tundish bottom	*B_1_*	m	0.78	0.459
Inclination of side walls	*ϒ*		7°	7°
Tundish filling level	*H*	m	0.8	0.471
Length of the shroud	*L_sh_*	m	1	1
Inner diameter of the shroud	*D_sh_*	m	0.068	0.04
Length of the SEN	*L_SEN_*	m	1	1
Inner diameter of the SEN	*D_SEN_*	m	0.07	0.04
Position of the SEN	*L_SEN,P_*	m	2.885	-
Immersion depth of the shroud	*Z_Sh_*	m	0.6	0.381
Diameter of the stopper rod	*d_sr_*	m	0.127	0.08
Fluid density	*ρ*	kg/m^3^	−0.883T + 8612.4	998.2
Dynamic viscosity	*μ*	Pa·s	5.975 × 10^−3^	1.008 × 10^−3^
Mass flow rate during steady-state casting	*m_sh,SEN_*	kg/s	38	3.68
Mean flow velocity inside the shroud	*u_sh_*	m/s	1.49	2.92
Theoretical mean flow velocity	$\bar{u}$	m/s	0.008	0.015
Maximum back-flow velocity in the tundish	*u*	m/s	-	0.07
Theoretical residence time of the fluid	*T_theo_*	s	420	126
Reynolds number	*Re*	*-*	10,380	10,380

**Table 2:** Total mesh number of CFD model.

Mesh Size (m)	0.0045	0.005	0.0055	0.006	0.0065	0.007
No. of mesh (million)	0.97	0.75	0.62	0.53	0.45	0.42

**Table 3:** Summary of user submissions.

User	Code	Turb. ^1^	Model	No. of Cells × 10^3^	Mesh ^2^	Solver Type &Precision ^3^	Free-Surface	Wall	Discretization	Processing Time (h)			
										Pre	Cal	Post	Total
1	FLUENT	RKE	Full	491	Hex.	Seg./Double	Symmetry	No-slip	2nd order	8	24	20	52
2	FLUENT	RSM	Full	560	Hex.	Seg./Double	Shear = 0	No-slip	QUICK	8	90	8	106
3	Fastest3D	SKE	Full	661	Hex.	Seg./Double	Wall	No-slip	1st, 2nd order	16	30	4	50
4	FLUENT	RKE	Full	540	Hex.	Seg./Single	Symmetry	No-slip	2nd order	18	24	6	48
5	CFX	SKW	Full	500	Tet.	Cou./single	Shear = 0	No-slip	2nd order	24	2	32	58
6	OpenFoam	RSM	Full	503	Hex.	Seg./Double	Shear = 0	No-slip	1st order	1.5	48	2	51.5
7	FLUENT	RSM	Full	556	Hex.	Seg./single	Symmetry	No-slip	2nd order	3	1	10	14
8	OpenFoam	RKE	Full	642	Hex.	Seg./Double	Symmetry	No-slip	1st, 2nd order	0.5	48	2	50.5
9	FLUENT	RSM	Full	384	Hex.	Seg./Double	Symmetry	No-slip	2nd order	4	9	3	16
10	FLUENT	RKE	Full	592	Hex.	Seg./Double	Wall	No-slip	1st, 2nd order	8	24	20	52
12	STAR-CCM+	RKE	Half	530	Pol.	Seg./Double	Wall	No-slip	2nd order	10	35	12	57

^1^ Turb.: turbulence model; RKE: realizable k-ε; RSM: Reynolds stress model; SKE: Standard k-ε; SKW: Shear stress transport k-ω; ^2^ Hex.: hexahedrons; Tet.: tetrahedrons: Pol.: polyhedral; ^3^ Seg.: segregated; Cou: coupled.

## Data Availability

The data presented in this study are available on request from the corresponding author.
